# Bioinformatics analysis of microarray data to reveal the pathogenesis of diffuse intrinsic pontine glioma

**DOI:** 10.1186/s40659-018-0175-6

**Published:** 2018-08-20

**Authors:** Li Wei, Fei He, Wen Zhang, Wenhua Chen, Bo Yu

**Affiliations:** 1 Department of Rehabilitation, Shanghai General Hospital, Shanghai Jiao Tong University, No. 100, Haining Road, Shanghai, 200080 China; 2 School of International Medical Technology, Shanghai Sanda University, No. 2727, Jinhai Road, Shanghai, 201209 China

**Keywords:** Diffuse intrinsic pontine glioma, Differentially-expressed genes, Neuroactive ligand–receptor interaction, Protein–protein interaction network, Transcription factor–microRNA–target gene network

## Abstract

**Background:**

Diffuse intrinsic pontine glioma (DIPG) is the main cause of pediatric brain tumor death. This study was designed to identify key genes associated with DIPG.

**Methods:**

The gene expression profile GSE50021, which consisted of 35 pediatric DIPG samples and 10 normal brain samples, was downloaded from the Gene Expression Omnibus database. Differentially expressed genes (DEGs) were identified by limma package. Functional and pathway enrichment analyses were performed by the DAVID tool. Protein–protein interaction (PPI) network, and transcription factor (TF)–microRNA (miRNA)–target gene network were constructed using Cytoscape. Moreover, the expression levels of several genes were validated in human glioma cell line U251 and normal glia HEB cells through real-time polymerase chain reaction (PCR).

**Results:**

A total of 378 DEGs were screened (74 up-regulated and 304 down-regulated genes). In the PPI network, GRM1, HTR2A, GRM7 and GRM2 had higher degrees. Besides, *GRM1* and *HTR2A* were significantly enriched in the neuroactive ligand–receptor interaction pathway, and calcium signaling pathway. In addition, *TFAP2C* was a significant down-regulated functional gene and *hsa*-*miR*-*26b*-*5p* had a higher degree in the TF-miRNA-target gene network. PCR analysis revealed that *GRM7* and *HTR2A* were significantly downregulated while *TFAP2C* was upregulated in U251 cells compared with that in HEB cells (p < 0.001). *GRM2* was not detected in cells.

**Conclusions:**

*GRM1* and *HTR2A* might function in DIPG through the neuroactive ligand–receptor interaction pathway and the calcium signaling pathway. Furthermore, the *TFAP2C* and *hsa*-*miR*-*26b*-*5p* might play important roles in the development and progression mechanisms of DIPG.

## Background

Diffuse intrinsic pontine glioma (DIPG) is the most common brain tumor in childhood [1]. The mortality of DIPG goes up with no available treatment, almost 100% fatality [2]. Although DIPG can be treated by radiotherapy and chemotherapy, the average survival time has remained only 9 months and 5-year survival time is less than 1% [3]. There were no obviously advantages of radiation and chemotherapy [4]. The development of therapies for DIPG was greatly hampered because of lack of therapeutic benefits and molecular studies [5]. Therefore, a better understanding of the molecular mechanisms underlying DIPG is helpful to develop new therapies for this disease.

In the past few years, DIPG cell cultures and orthotopic xenograft models have been established [6, 7]. Previous study showed that grade of gliomas in human brain was related to the *R*-*Ras* expression and phosphorylation, indicating the *EphB2/R*-*Ras* signaling pathway as a potential target associated with cell adsorption, growth and invasion [8]. Holland et al. [9] found that activation of *Ras* and *Akt* in neural progenitor cells can induce glioma in mice, and the *Ras* and *Akt* proteins play important roles in the pathogenesis of gliomas. These studies suggest that a single gene or the interaction between more genes involved in the promotion of disease occurrence and development. In recent years, the advantage of gene chip technology and bioinformatics analysis is obviously observed, which is applied to analyze the molecular mechanism of DIPG [10]. Deng et al. [10] showed cholecystokinin (*CCK*) and gastrin (*GAST*) associated with the G-protein coupled receptor (*GPCR*) signaling pathway, and 5-hydroxytryptamine (serotonin) receptor 7 (*HTR7*) involved in the neuroactive ligand–receptor interaction might play critical roles in DIPG. Despite a number of researches have investigated the molecular basis of DIPG, the molecular mechanisms of the disease remain not fully understood.

In the study of Buczkowicz et al. [2], the gene expression profile GSE50021 was utilized only for surveying what urged DIPGs by whole-genome sequencing. In the study of Deng et al. [10], GSE50021 was analyzed and revealed a potential key molecular mechanisms in DIPG by microarray analysis and bioinformatics analysis. Recently, Xi et al. [11] used a novel method for extracting DEGs from GSE50021 in combination with GSE50022 that included DNA methylation. However, given the complicated molecular mechanisms of DIPG, it is necessary to fully utilize GSE50021 profile to identify more potential genes and pathways related to DIPG. In this study, differentially-expressed genes (DEGs) were indentified from GSE50021 dataset. Subsequently, enrichment analysis, protein–protein interaction (PPI) network, module analyses, and microRNAs (miRNAs)–transcription factors (TFs)–target gene regulatory network analysis were successively performed to identify the key genes implicated in the pathogenesis of DIPG. Importantly, several key genes were validated through real-time polymerase chain reaction (PCR).

## Methods

### Microarray data

The gene expression profile GSE50021 was downloaded from the Gene Expression Omnibus (http://www.ncbi.nlm.nih.gov/geo/) database [12], which was based on the platform of GPL13938 Illumina HumanHT-12 WG-DASL V4.0 expression beadchip. This dataset, including 35 DIPG samples and 10 normal brain samples, was deposited by Buczkowicz et al. [2].

### Data preprocessing and DEGs screening

Using robust multi-array average (RAM) [13] method of Affy package (http://www.bioconductor.org/packages/release/bioc/html/affy.html) in R language, the raw data were preprocessed, including background correction, normalization and expression calculation. The platform annotation file was used to annotate the probes, and the probes without corresponding gene symbols were removed. For different probes mapped to the same gene, the average value of the probes was taken as the final gene expression value. DEGs were identified by the classical Bayes method in Limma package [14] (http://www.bioconductor.org/packages/2.9/bioc/html/limma.html). The genes with adjusted *p*-value < 0.05 were chosen as DEGs.

### Functional and pathway enrichment analyses

Gene Ontology (GO) (http://www.geneontology.org) analysis [15], including biological process (BP), molecular function (MF), and cellular component (CC), is used for functional study of single gene or large-scale genome. The Kyoto Encyclopedia of Genes and Genomes (KEGG, http://www.genome.ad.jp/kegg/) [16, 17] is the major recognized pathway-related database, which takes into account not only each KEGG pathway itself, but also its related pathways [17]. The DAVID online tool (https://david-d.ncifcrf.gov/) [18] was used to perform GO functional and KEGG pathway enrichment analysis for the DEGs. The *p*-value of < 0.05 and gene count ≥ 2 were chosen as the significant thresholds. In order to directly observe the functions of DEGs, the ClueGO plug-in [19, 20] http://apps.cytoscape.org/apps/ClueGO) of Cytoscape [21] was applied to visualize the results of enrichment analysis in figures, and *p*-value of < 0.05 was chosen as the significant threshold.

### PPI network and module analyses

The Search Tool for the Retrieval of Interacting Genes (STRING) [22] (http://www.string-db.org/) is an online database providing experimental and predicted PPI information. In this study, the STRING database [22] was used to analyze the PPIs among the proteins encoded by the DEGs with a combined score of > 0.4, then the PPI networks for the up-regulated and the down-regulated genes were separately visualized by Cytoscape software (http://www.cytoscape.org/) [21]. The CytoNCA plug-in [23] (http://apps.cytoscape.org/apps/cytonca) in Cytoscape software was used to analyze the topological property of the network, acquiring the important nodes in the PPI network combined with the degree of each node.

In addition, module analysis was performed for the PPI networks using the MCODE plug-in (http://apps.cytoscape.org/apps/mcode) [24] in Cytoscape software. An adjusted p < 0.01 was chosen as the significance threshold. In addition, the nodes in the significant modules were performed GO functional and KEGG pathway enrichment analyses using DAVID online tool [18].

### Construction of TF-miRNA-target gene regulatory network

At the post-transcription stage, miRNAs regulate gene expression [25]. Whereas, TFs can promote or repress transcription at a pre-transcription stage [26]. TF-miRNA-target gene acts as a tumor suppressor network, triggering a comprehensive change in genetic programs involving cell proliferation, apoptosis and cancer invasion in cancer [27]. The miRNAs associated with DIPG and their target genes were searched using miRWalk2.0 database [28] (http://zmf.umm.uni-heidelberg.de/apps/zmf/mirwalk2/). Through comparing target genes with the DEGs, miRNA-DEG pairs were obtained. Then, miRNA-DEG regulatory network was visualized by Cytoscape software [21].

The iRegulon plug-in [29] (http://apps.cytoscape.org/apps/iRegulon) in Cytoscape software, which included the TF-target pairs of multiple human databases such as Transfac (http://www.gene-regulation.com/pub/databases.html) [30], and Encode (https://www.encodeproject.org/) [31, 32], was used to predict the TF-target pairs in the miRNA-DEG regulatory network, A normalized enrichment score (NES) > 3 was chosen as the significant threshold for screening TF-target pairs. Additionally, the TF-miRNA-target regulatory network was visualized using Cytoscape software [21].

### Real-time PCR verification of the expression of key genes

Human glioma cell line U251, a common used DIPG cell line [33], was purchased from cell bank of Chinese Academy of Sciences (Shanghai, China) and normal glia HEB cells [33] were purchased from GuangZhou Jennio Biotech Co., Ltd, Guangdong, China. All cells were grown in Dulbecco’s modified eagle medium supplemented with 10% fetal bovine serum and 1% antibiotics (penicillin and streptomycin) at 37  °C in an atmosphere of 10% CO_2_.

Briefly, total RNAs were isolated from 5 × 10^6^ to 10 × 10^6^ cell samples using a TRIzol reagent (Invitrogen, CA, USA). RNA concentration and quality were determined using a TECAN infinite M100 PRO Biotek microplate reader (TECAN, CA, USA). Total RNA (0.5 μg) was used for cDNA synthesis using the PrimeScript RT Master Mix (RR036A; Takara, Dalian, China). PCR was performed using the SYBR GREEN kit (4367659; Thermo, USA) in Viia7 Real-Time PCR System (Applied Biosystems, USA). The primers used in this study are listed in Table 1.

**Table 1:** The primers used in real time PCR

Primer name	Sequences (5′-3′)
GRM2-hF	GCTCCACTCCGATTCTCTCC
GRM2-hR	GAAGCAGCGAAGGCAAAGAG
GRM7-hF	GACACTTACGCGCTCGAACA
GRM7-hR	TCATCACTTAGCTCGGGTGC
HTR2A-hF	CTGGTCTGCTTTACTGACAGCC
HTR2A-hR	AGAGCACGTCCAGGTAAATCC
GAPDH-hF	TGACAACTTTGGTATCGTGGAAGG
GAPDH-hR	AGGCAGGGATGATGTTCTGGAGAG

### Statistical analysis

Data are presented as mean ± standard deviation. Statistical analysis was performed using SPSS 22.0 (IBM, Armonk, NY, USA). Differences in gene expression levels between groups were analyzed by one-way analysis of variance. The p < 0.05 was considered significant.

## Results

### DEGs screening

**Fig. 1:**
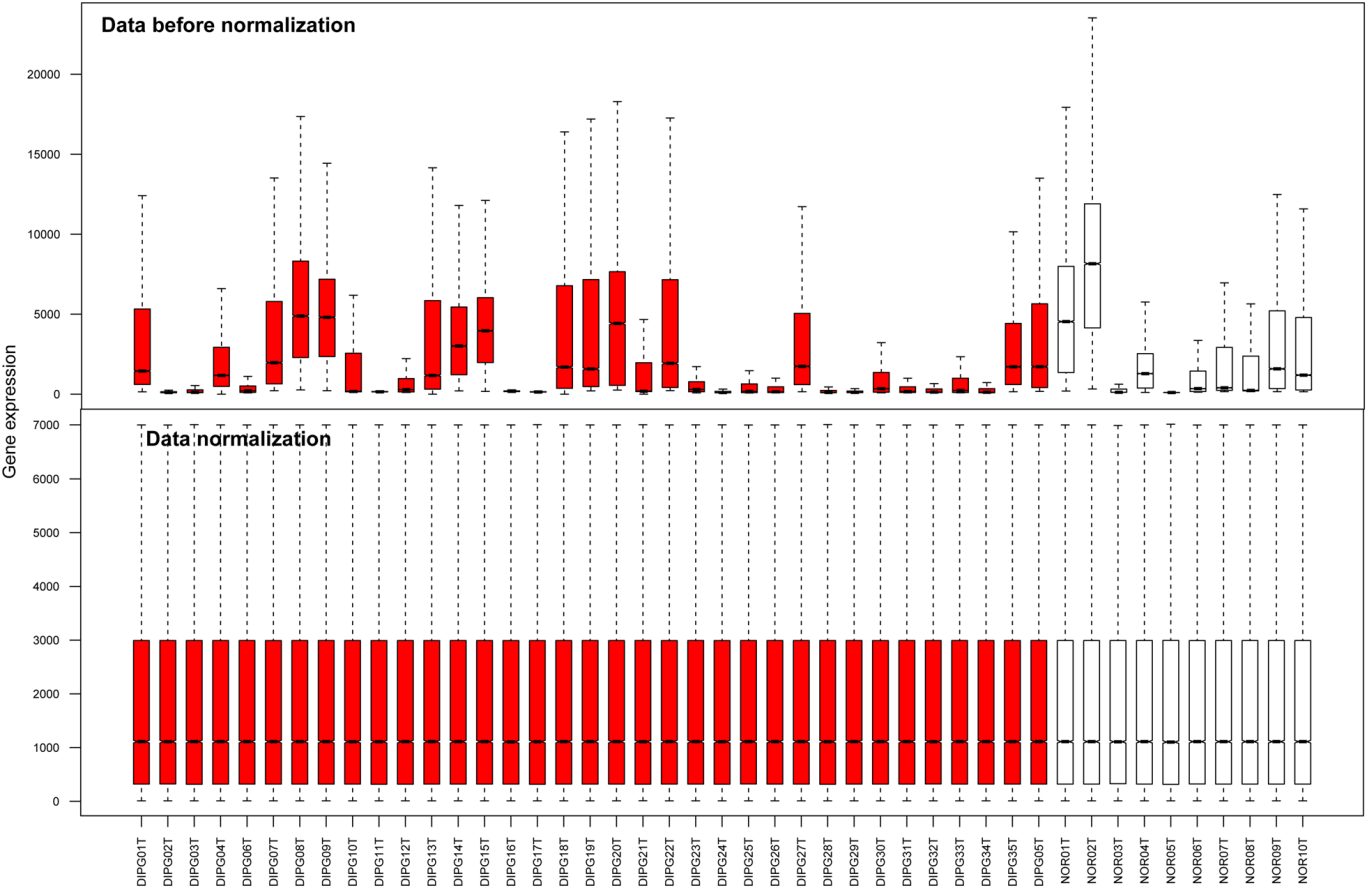
The box figures before and after normalization. Red and white separately represent the diffuse intrinsic pontine glioma (DIPG) samples and the normal samples

As shown in Fig. 1, the medians located at the same level after performing data normalization, which indicated a perfect effect. Based on adjusted *p*-value < 0.05, 378 DEGs were identified, which included 74 up-regulated and 304 down-regulated genes.

### Functional and pathway enrichment analyses

**Fig. 2:**
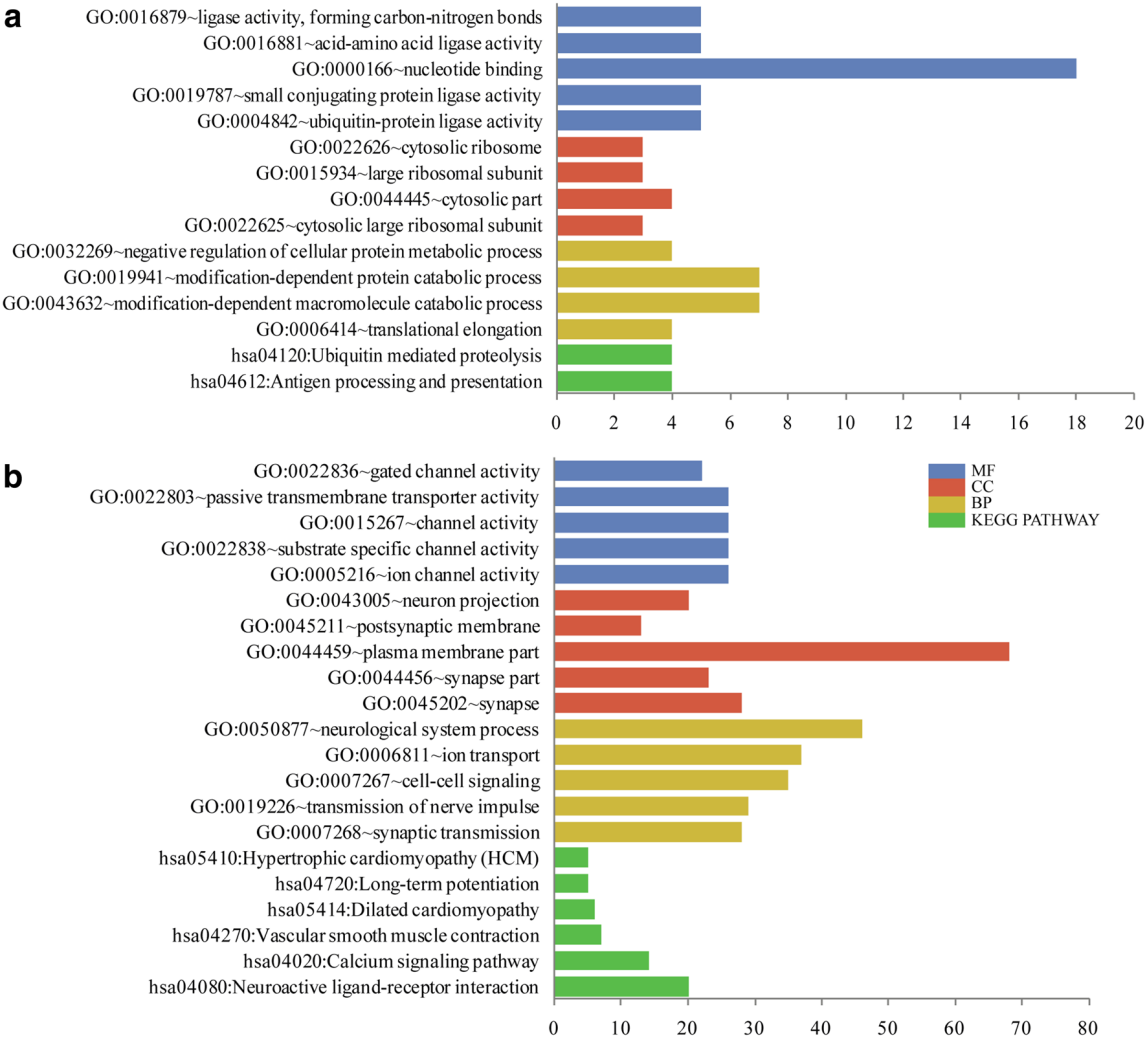
The top five GO terms and KEGG pathways enriched separately for the up-regulated genes (**a**) and the down-regulated (**b**). *MF* molecular function, *BP* biological processes, *CC* cellular components, *KEGG* Kyoto Encyclopedia of Genes and Genomes, *GO* gene ontology; The horizontal axis represents the count of enriched DEGs. The vertical axis represents the enriched GO terms and KEGG pathways

According to the *p*-values (ascending sort), the top five enriched terms are exhibited in Fig. 2. The up-regulated genes were significantly enriched in the modification-dependent macromolecule catabolic process (BP, *p* = 0.044), nucleotide binding (MF, *p* = 0.007), cytosolic part (CC, *p* = 0.044), and antigen processing and presentation (pathway, *p* = 0.01) (Fig. 2a). While the down-regulated genes were significantly associated with neurological system process (BP, *p* = 2.33E−08), ion channel activity (MF, *p* = 6.64E−09), plasma membrane part (CC, *p* = 1.48E−08), neuroactive ligand–receptor interaction (pathway, *p* = 1.57E−08) and calcium signaling pathway (pathway, *p* = 4.18E−06) (Fig. 2b).

**Fig. 3:**
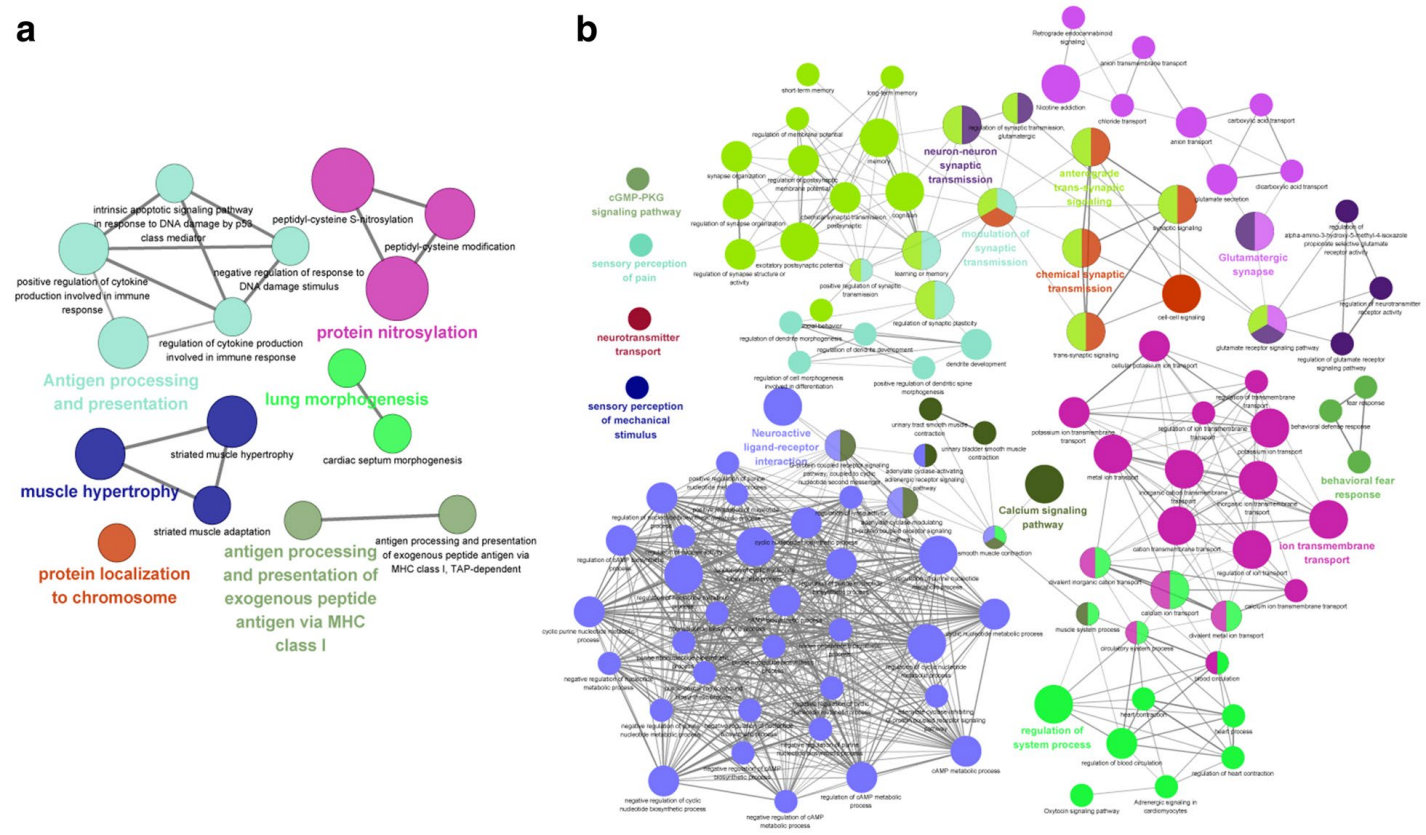
The crosslinking enrichment of GO-BP terms and KEGG pathways enriched separately for the up-regulated genes (**a**) and the down-regulated (**b**). The nodes with different color represent different GO-BP or KEGG pathways. *GO* gene ontology, *BP* biological processes, *KEGG* Kyoto Encyclopedia of Genes and Genomes

Furthermore, the crosslinking enrichment of GO-BP terms and KEGG pathways are shown in Fig. 3. The more down-regulated genes were related to the disease, and the main pathway was neuroactive ligand–receptor interaction (Fig. 3).

### PPI network and module analyses

**Fig. 4:**
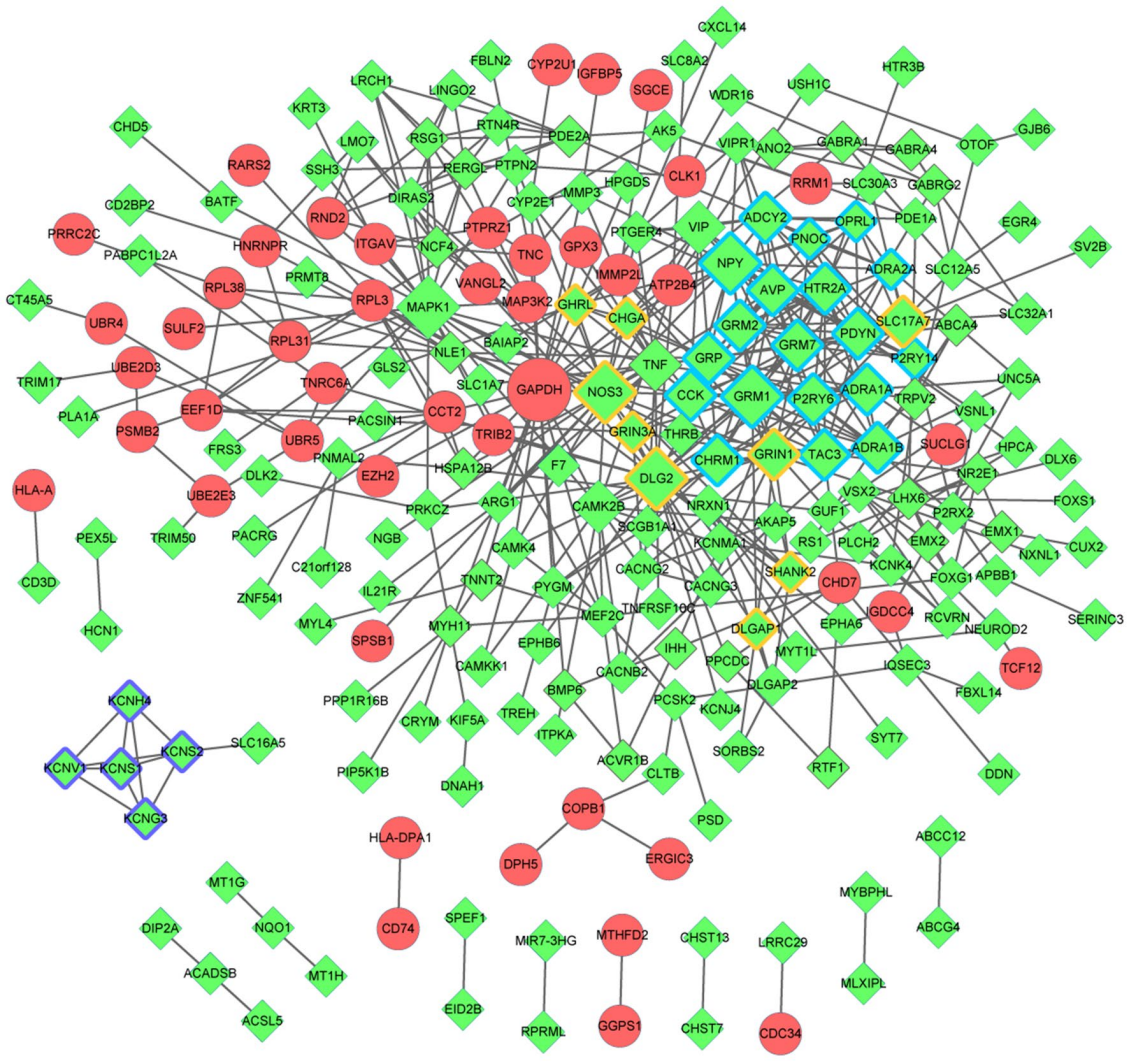
Protein–protein interaction network constructed for the DEGs. The red circle and the green rhombus represent up-regulated genes and down-regulated genes, respectively. The nodes in Module 1, Module 2 and Module 3 separately were marked by blue, purple and yellow edges. *DEGs* differentially-expressed genes

Based on the STRING database, the PPI network for the DEGs (including 231 nodes and 490 edges) was constructed (Fig. 4). Up-regulated gene with higher node degree was glyceraldehyde-3-phosphate dehydrogenase (*GAPDH*). Down-regulated genes had higher degrees were as follows: nerve peptide Y (*NPY*), 5-hydroxytryptamine receptor 2A (*HTR2A*), metabotropic glutamate receptor 1 (*GRM1*), adenylate cyclase 2 (*ADCY2*), *GRM2*, *GRM7* and so on. The nodes with degree ≥ 10 are listed in Table 2.

**Table 2:** The differentially-expressed genes (DEGs) with a degree > 10 in the protein–protein interaction (PPI) network

Gene	Description	Degree	Gene	Description	Degree	Gene	Description	Degree
*GAPDH*	Up	27	*CAMK2B*	Down	16	*HTR2A*	Down	12
*MAPK1*	Down	23	*AVP*	Down	16	*TAC3*	Down	12
*NPY*	Down	22	*TNF*	Down	16	*VIP*	Down	11
*DLG2*	Down	22	*CCK*	Down	15	*ADRA1A*	Down	11
*NOS3*	Down	20	*GRIN1*	Down	15	*CHRM1*	Down	11
*GRM1*	Down	20	*GRM7*	Down	15	*ADRA1B*	Down	10
*GRP*	Down	16	*ADCY2*	Down	14	*P2RY6*	Down	10
*PDYN*	Down	16	*GRM2*	Down	14	*SLC17A7*	Down	10

Additionally, three significant modules, including module 1 (19 nodes and 95 edges), module 2 (5 nodes and 10 edges) and module 3 (9 nodes and 15 edges), were acquired by MCODE plug-in (Fig. 4).

Furthermore, 10 KEGG pathways were significantly enriched by module 1, including neuroactive ligand–receptor interaction (*p* = 7.94E−12), calcium signaling (*p* = 1.83E−05), glutamatergic synapse (*p* = 1.39E−03). Meanwhile, the nodes in module 3 were significantly enriched in four KEGG pathways, including cocaine addiction (*p* = 2.37E−06), nicotine addiction (*p* = 6.74E−04), cAMP signaling pathway (*p* = 1.56E−02) and glutamatergic synapse (*p* = 4.86E−02) (Table 3). However, no pathways were enriched for the nodes in module 2.

**Table 3:** The enriched pathways for the nodes in module 1 and 3

Pathway name	Count	*p*-value	Genes
Module 1			
hsa04080:neuroactive ligand–receptor interaction	11	7.94E−12	*P2RY6, GRM2, OPRL1, P2RY14, CHRM1, GRM7, ADRA2A, ADRA1B, ADRA1A, GRM1, HTR2A*
hsa04020:calcium signaling pathway	6	1.83E−05	*ADCY2, CHRM1, ADRA1B, ADRA1A, GRM1, HTR2A*
hsa04724:glutamatergic synapse	4	1.39E−03	*ADCY2, GRM2, GRM7, GRM1*
hsa04022:cGMP-PKG signaling pathway	4	4.08E−03	*ADCY2, ADRA2A, ADRA1B, ADRA1A*
hsa04970:salivary secretion	3	1.26E−02	*ADCY2, ADRA1B, ADRA1A*
hsa04540:gap junction	3	1.32E−02	*ADCY2, GRM1, HTR2A*
hsa04270:vascular smooth muscle contraction	3	2.34E−02	*ADCY2, ADRA1B, ADRA1A*
hsa04261:adrenergic signaling in cardiomyocytes	3	3.42E−02	*ADCY2, ADRA1B, ADRA1A*
Module 3			
hsa04724:glutamatergic synapse	5	2.37E−06	*SLC17A7, DLGAP1, GRIN1, GRIN3A, SHANK2*
hsa05033:nicotine addiction	3	6.74E−04	*SLC17A7, GRIN1, GRIN3A*
hsa04024:cAMP signaling pathway	3	1.56E−02	*GRIN1, GHRL, GRIN3A*
hsa05030:cocaine addiction	2	4.86E−02	*GRIN1, GRIN3A*

### TF-miRNA-target regulatory network analysis

**Fig. 5:**
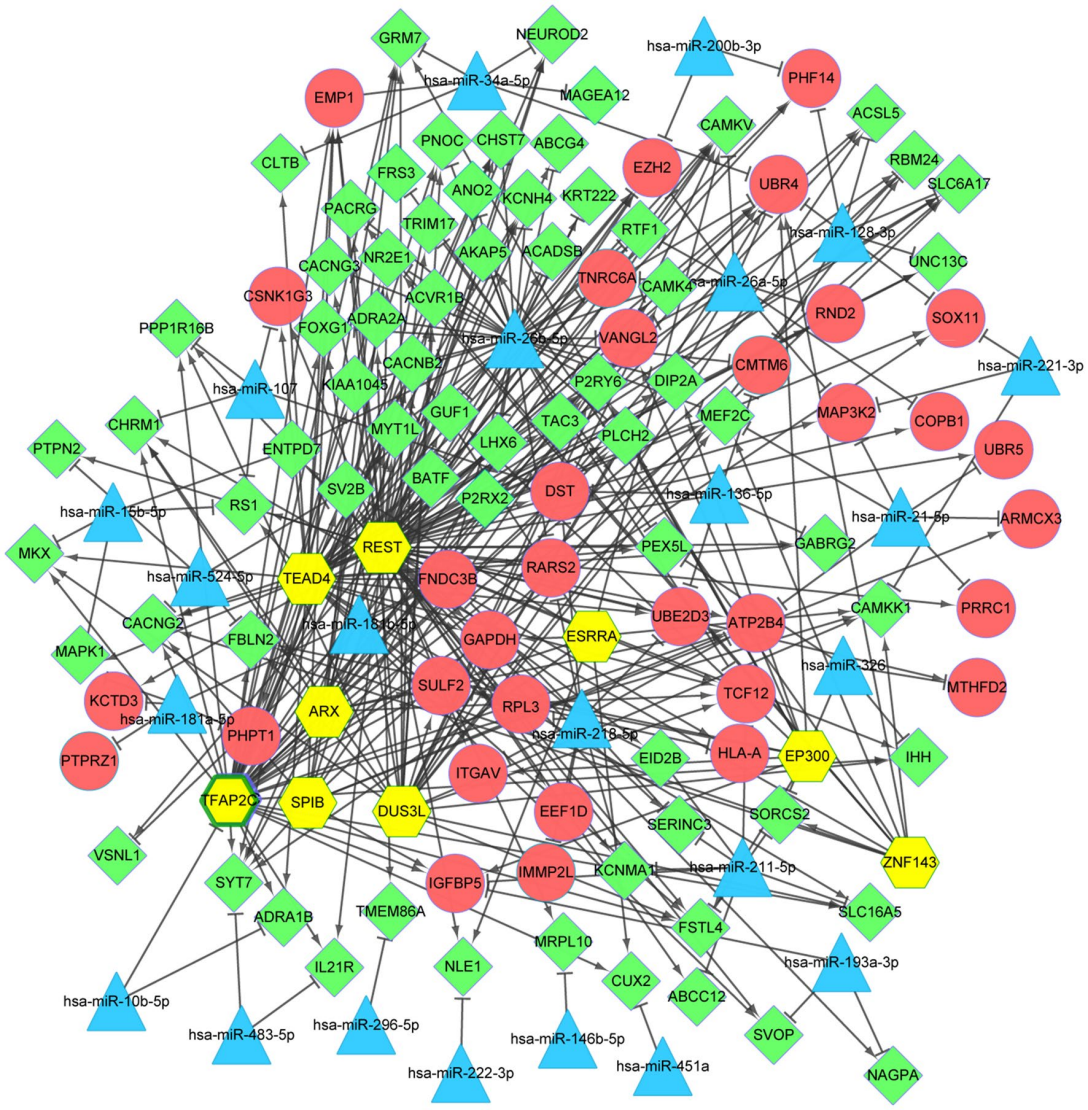
The miRNA-TF-target regulatory network. The red circle and the green rhombus represent up-regulated genes and down-regulated genes, respectively. The blue triangle represents miRNA, and yellow hexagon indicates TFs. T shape represents the miRNA-target relationship, and arrow represents TF-target relationship. *DEGs* differentially-expressed genes, *TFs* transcription factors, *miRNAs* microRNAs

A total of 36 miRNAs associated with DIPG were identified from miRWalk2.0, and only 27 miRNAs remained after removing the repeats and the miRNAs in mice. The target genes of the remaining miRNAs were compared with the DEGs and a total of 141 miRNA-DEG pairs were obtained. The miRNA-DEG regulatory network was visualized by Cytoscape software, consisting of 136 nodes and 368 edges (Fig. 5). Based on the iRegulon plug-in, a total of nine TFs were identified from the miRNA-DEG regulatory network. Then, the TF-miRNA-target regulatory network was constructed and the nodes with top 10 degrees are listed in Table 4. The *TFAP2C* was a significant down-regulated functional gene, while the *hsa*-*miR*-*26b*-*5p* had a higher degree in the TF-miRNA-target regulatory network.

**Table 4:** The differentially-expressed genes (DEGs) with a degree > 10 in the transcription factors–microRNAs–target regulatory network

Gene	Description	Degree	Gene	Description	Degree
*REST*	TF	71	*hsa*-*miR*-*218*-*5p*	miRNA	15
*hsa*-*miR*-*26b*-*5p*	miRNA	41	*SPIB*	TF	12
*TFAP2C*	TF/down	39	*EP300*	TF	12
*TEAD4*	TF	36	*hsa*-*miR*-*26a*-*5p*	miRNA	11
*DUS3L*	TF	24	*ZNF143*	TF	10
*ARX*	TF	15	*UBR4*	Up	10

### Real-time PCR verification of the expression of key genes

**Fig. 6:**
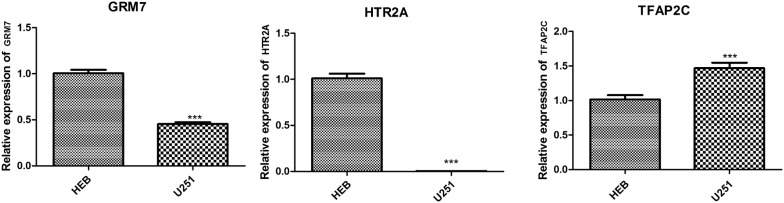
The gene expression levels of *GRM7*, *HTR2A* and *TFAP2C* detected by real-time polymerase chain reaction. ***p < 0.001 compared with control

Expression levels of *GRM2*, *GRM7*, *HTR2A* and *TFAP2C* were determined using real-time PCR. As shown in Fig. 6, *GRM7* and *HTR2A* were significantly downregulated while *TFAP2C* was significantly upregulated in U251 cells compared with that in HEB cells (p < 0.001). *GRM2* was not detected in cells, which may be due to its expression level being too low in them or the difference between tissue and cell samples.

## Discussion

In order to identify potential genes and related pathways of DIPG, a further analysis based on bioinformatics method was performed on the GSE50021 profile. Deng et al. [10] also used GSE50021 profile to analyze candidate genes and associated pathways, and identified 679 DEGs (454 up-regulated and 225 down-regulated genes) but the results had both similarities and dissimilarities on the number and function of DEGs. In the present study, 378 DEGs were identified, including 74 up-regulated genes and 304 down-regulated genes. The difference on the number of DEGs is due to the different thresholds of DEGs screening. In the study of Deng et al. [10], genes with *p*-value of < 0.01 and |log2fold change (FC)| > 2.0 were selected as DEGs for further study, while our study used adjusted *p*-value < 0.05 as threshold. Based on the module analysis, the down-regulated genes were significantly enriched in different GO terms and pathways, for instance, the *GRM1* and *HTR2A* were associated with neuroactive ligand–receptor interaction and calcium signaling pathway, while the *ADCY2* was associated with calcium signaling pathway, in addition, *NPY* with higher degree in PPI network. TF-miRNA-target gene network showed that *TFAP2C* was a significantly down-regulated functional gene and the *hsa*-*miR*-*26b*-*5p* had a higher degree.

*GRM1*, *GRM2* and *GRM7* had higher degrees in module 1, which were enriched with neuroactive ligand–receptor interaction, calcium signaling pathway and glutamatergic synapse in KEGG pathways in this study. Chen et al. [34] demonstrated that neuroactive ligand–receptor interactions are mainly associated with DIPG. Calcium is an essential signal transduction element that regulate numerous eukaryotic cellular functions including cell cycle progression [35]. Deregulation of the calcium signalling is linked to each of the ‘cancer hallmarks’ [36]. Additionally, the expression of glutamatergic system is implicated in tumour biology [37]. Given the role of these pathways in cancers, we speculated that *GRM1*, *GRM2* and *GRM7* may be associated with DIPG progression by involving in these pathways.

*GRM1*, *GRM2* and *GRM7* belong to the glutamate receptor family, which included ionotropic glutamate receptors (iGluR) and metabotropic glutamate receptors (mGluR) [38]. The mGluRs are further divided into three groups, among which, GRM1 is owned by group I, while GRM2 and GRM7 belong to group II and III respectively [39]. Emerging evidence has suggested a role for glutamate and its receptors in the biology of cancer. Glutamate receptor antagonists could limit tumor growth [40]. Blocking expression of selected GluR subunits inhibits proliferation of cancer cells in vitro [41]. Importantly, it has been demonstrated that glutamate receptor subunits are expressed in a variety of tumors, including glioma [42]. Previous studies reported that the aberrant expression of *GRM1* induced spontaneous melanoma development in vivo [43, 44]. Brocke et al. [45] demonstrated that tumor growth may be suppressed via interfering with glutamate signaling, and suggested that glutamate receptor modulators may be an adjunctive treatment for central nervous system tumors. The study has shown that the *GRM2* had huge potential for treating psychiatric and neurological diseases throughout the mammalian central nervous system, and that have been proposed as major targets for the development of drugs for human psychiatric and neurological diseases [46]. Recently, Ma et al. [47] reported that GRM2 was downregulated in glioma cells, and was regulated by eight transcription factors. In our study, *GRM2* had higher degree in the PPI network. Previous study has reported that hubs that are “highly connected” in a PPI network tend to correspond to essential genes, which is called the “centrality–lethality rule” [48]. All these results may suggest the essential role of *GRM2* in glioma. Furthermore, De et al. [49] revealed that *GRM7* was associated with mood disorders, suicide, and treatment response. *GRM7* has been reported to be hypermethylated in breast cancer cells [50]. Specially, the expression level of *GRM7* was validated in human glioma cell line U251. Taken together, these suggested that the *GRM1*, *GRM2* and *GRM7* might function in DIPG.

In this study, *HTR2A* had a higher degree and was strongly related with neuroactive ligand–receptor interactions. Besides, it was demonstrated to be down-regulated in U251 cells. Li et al. [51] have demonstrated that *HTR2A* are related with low-grade and high-grade gliomas via neuroactive ligand–receptor interactions. Thus, the present results suggested that *HTR2A* might play an important role in gliomas via neuroactive ligand–receptor interactions, which was consistent with previous findings. Besides, *HTR2A* was also enriched in calcium signaling pathway, meanwhile, *ADCY2* was significantly related with the calcium signaling pathway as well. Being consistent with these results, Deng et al. [10] demonstrate that *ADCY2* plays a role by the calcium signaling pathway in DIPG tumorigenesis. Hall et al. [52] showed that NPY-immunopositive played an important role in modulating cortical excitability of interneurons. NPY had a higher degree in the PPI network, suggesting that *NPY* might function in CNS.

It has been well known that TFs promote or repress transcription at a pre-transcription stage [26], while miRNA plays an important regulatory roles at the post-transcriptional level [25]. Many previous studies have investigated the pathogenesis of DIPG from transcriptional level using glioma cell line U251 [53–55]. In the present study, unlike the other studies using GSE50021 for analysis, we performed miRNA-TF-target gene regulatory network analysis in order to predict the TFs and miRNAs that may play roles in DIPG. *TFAP2C* was identified as candidate TF, which was a significantly down-regulated functional gene. However, result of PCR analysis showed that *TFAP2C* was upregulated in human glioma cell line U251, which was inconsistent with the prediction result. The contradiction may be due to the heterogeneity between tissue samples and cell samples. Further experiment is needed to detect the expression level of *TFAP2C* in DIPG. Study has suggested that *TFAP2C* promotes lung tumorigenesis by up-regulation of TGFBR1 and consequent activation of PAK1 signaling [56]. Another study showed that through regulation of *RET*, the expression of *TFAP2C* decreased in luminal breast cancer. Besides, EGFR and HER2 were regulated by *TFAP2C* in breast cancer [57]. Therefore, *TFAP2C* may play a role in cancer. The *hsa*-*miR*-*26b*-*5p* had a higher degree in the TF-miRNA-target regulatory network, which may be related to DIPG. The research indicated that proliferation and apoptosis in lung cancer cells were inhibited via a *miR*-*26b*-*5p*-EZH2-mediated approach [58]. Although the roles of *TFAP2C* and *hsa*-*miR*-*26b*-*5p* in DIPG have not been discussed, we inferred that *TFAP2C* and *hsa*-*miR*-*26b*-*5p* may play roles in DIPG tumorigenesis. Further genetic studies are required to verify this hypothesis.

Specially, the present study used real-time PCR to verify the expression of key genes *GRM2*, *GRM7*, *HTR2A* and *TFAP2C* through human glioma cell line U251. *GRM7* and *HTR2A* were significantly downregulated in U251 cells compared with that in HEB cells, which was in accordance with the analysis results. *GRM2* was not detected in cells, which may be due to its expression level being too low in U251 cells or the difference between tissue and cell samples. *TFAP2C* was upregulated in human glioma cell line U251, being inconsistent with the prediction result, which needed to be further investigated. In the study of Buczkowicz et al. [2], the data was obtained from tumor tissue biopsy vs. normal. It is possible that different cell types contribute to gene expression change, therefore it is really a limitation that only one cell line was used for gene validation. We will continue collecting tissue samples for validation in the future.

In conclusion, this study indicated that *GRM1*, *GRM7* and *HTR2A* might function in DIPG through the calcium signaling pathway and the neuroactive ligand–receptor interaction pathway. Meanwhile, *TFAP2C* and *hsa*-*miR*-*26b*-*5p* might have critical roles in the tumorigenesis of DIPG. This study provides new insights into the molecular mechanisms for the progress of DIPG and suggests directions for future study.

### Highlights

1. A total of 378 differentially-expressed genes were identified.
2. We found 490 protein–protein interactions, 9 transcription factors and 27 microRNAs.
3. *GRM1*, *HTR2A*, *TFAP2C* and *hsa-miR-26b-5p* might be related to diffuse intrinsic pontine glioma.

## Abbreviations

DIPG: diffuse intrinsic pontine glioma
DEGs: differentially-expressed genes
PPI: protein–protein interaction
TF: transcription factor
RAM: robust multi-array average
BP: biological process
MF: molecular function
CC: cellular component
